# Bioinformatic analysis of ciliary transition zone proteins reveals insights into the evolution of ciliopathy networks

**DOI:** 10.1186/1471-2164-15-531

**Published:** 2014-06-26

**Authors:** Amy R Barker, Karen S Renzaglia, Kimberley Fry, Helen R Dawe

**Affiliations:** Biosciences, College of Life and Environmental Sciences, University of Exeter, Exeter, EX4 4QD UK; Department of Plant Biology, Southern Illinois University, Carbondale, IL 62901 USA

**Keywords:** (3–10), Ciliopathy, Cilia, Transition zone, Compartmentalisation, Permeability, MKS, NPHP, Evolution

## Abstract

**Background:**

Cilia are critical for diverse functions, from motility to signal transduction, and ciliary dysfunction causes inherited diseases termed ciliopathies. Several ciliopathy proteins influence developmental signalling and aberrant signalling explains many ciliopathy phenotypes. Ciliary compartmentalisation is essential for function, and the transition zone (TZ), found at the proximal end of the cilium, has recently emerged as a key player in regulating this process. Ciliary compartmentalisation is linked to two protein complexes, the MKS and NPHP complexes, at the TZ that consist largely of ciliopathy proteins, leading to the hypothesis that ciliopathy proteins affect signalling by regulating ciliary content. However, there is no consensus on complex composition, formation, or the contribution of each component.

**Results:**

Using bioinformatics, we examined the evolutionary patterns of TZ complex proteins across the extant eukaryotic supergroups, in both ciliated and non-ciliated organisms. We show that TZ complex proteins are restricted to the proteomes of ciliated organisms and identify a core conserved group (TMEM67, CC2D2A, B9D1, B9D2, AHI1 and a single TCTN, plus perhaps MKS1) which are present in >50% of all ciliate/flagellate organisms analysed in each supergroup. The smaller NPHP complex apparently evolved later than the larger MKS complex; this result may explain why RPGRIP1L, which forms the linker between the two complexes, is not one of the core conserved proteins. We also uncovered a striking correlation between lack of TZ proteins in non-seed land plants and loss of TZ-specific ciliary Y-links that link microtubule doublets to the membrane, consistent with the interpretation that these proteins are structural components of Y-links, or regulators of their formation.

**Conclusions:**

This bioinformatic analysis represents the first systematic analysis of the cohort of TZ complex proteins across eukaryotic evolution. Given the near-ubiquity of only 6 proteins across ciliated eukaryotes, we propose that the MKS complex represents a dynamic complex built around these 6 proteins and implicated in Y-link formation and ciliary permeability.

**Electronic supplementary material:**

The online version of this article (doi:10.1186/1471-2164-15-531) contains supplementary material, which is available to authorized users.

## Background

Eukaryotic cilia and flagella are critical for diverse functions, from motility to signal transduction. It is generally accepted that the last eukaryotic common ancestor (LECA) was flagellate [1, 2]. There have been numerous modifications to, and even loss of, cilia in certain lineages such as yeasts and higher plants; however, most of the extant eukaryotic supergroups contain ciliated members. The underlying ultrastructure of cilia/flagella are highly conserved; further, while cilia are made up of hundreds of proteins, many are conserved across eukaryotes [1, 3, 4].

In humans, ciliary dysfunction causes inherited diseases, termed ciliopathies, characterised by aberrant embryonic development typically leading to cystic renal disease and malformations of the skeleton and/or central nervous system [5, 6]. Many of the ciliopathy phenotypes have been linked to perturbation of cell signalling pathways during development, in particular the Hedgehog (Hh) and Wnt developmental signalling pathways. Cilia are required for Hh signalling in vertebrates [7–9], while the role of cilia in Wnt signalling is less well understood [10].

This requirement for cilia in developmental signalling highlights the importance of compartmentalisation in ciliary function. Unlike most organelles, the cilium is not completely membrane-bound; despite this, the composition of the ciliary membrane is distinct from that of the cell membrane [11]. Passage of signalling intermediates between the ciliary compartment and the cytoplasm is key to ciliary signalling, particularly Hh [9]. However, until very recently, the molecular mechanisms governing this compartmentalisation have remained unknown.

**Figure 1:**
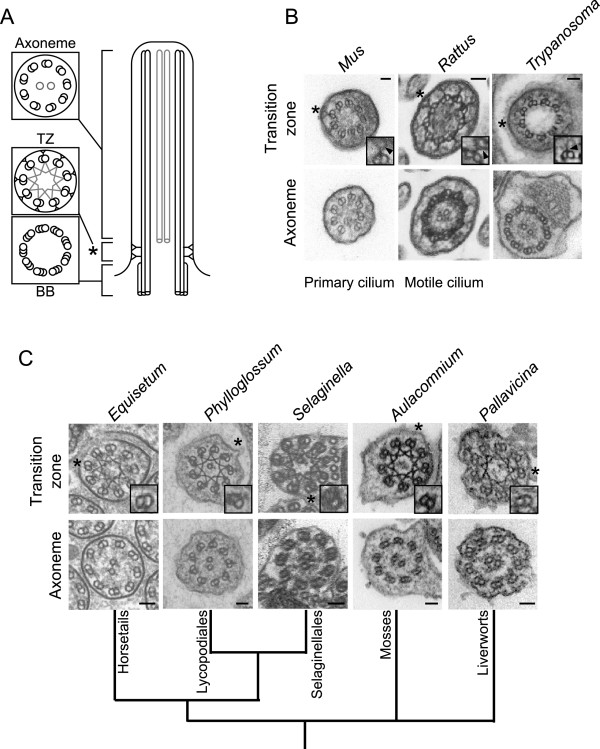
**Non-seed land plants lack ciliary Y-links. A** - Cartoon of cilium structure indicating the basal body (BB), transition zone (TZ) and axoneme. The central pair microtubules (present in motile cilia) and stellate structure (present in Plantae) are shown in grey. **B**, **C** - Transmission electron micrographs of axoneme and transition zone architecture in mammals/trypanosomes **(B)** and non-seed land plants **(C)**. Boxes represent a single enlarged doublet indicated by the asterisk; doublets were rotated to position the ciliary/flagellar membrane towards the top and right of the boxed area. Scale bars = 50 nm. Arrows indicate Y-links visible in mammalian and trypanosome cilia/flagella (**B**, top 3 panels). Note that these structures are absent in all non-seed plants examined (**C**, top 6 panels).

Several recent studies have implicated a number of mechanisms in formation of a ciliary ‘gate’ which controls entry and exit of proteins from the cilium, including a septin barrier [12], a nuclear pore-like mechanism [13–15], and retention of proteins via the apical actin cytoskeleton [16]. Several studies have highlighted complexes consisting largely of ciliopathy proteins which appear to be key in maintaining ciliary compartmentalisation [17–20]. These complexes localise to the ciliary transition zone (TZ), which is found distal to the basal body and, in motile cilia/flagella, ends at the basal plate where the central pair microtubules are nucleated (Figure 1A). This region is characterised by projections (Y-links) connecting the microtubule doublets to the ciliary membrane.

The original studies identified separate but overlapping complexes localising to the TZ, disruption of which causes changes to ciliary permeability [17–20]. The current model proposes two modules; the MKS/TCTN/B9 complex and the NPHP complex [21, 22], which are linked in *C. elegans* by RPGRIP1L [20]. Given that several of these proteins contain transmembrane domains, and that disruption of TZ complex proteins often causes loss of ciliary Y-links [20, 23, 24], the model also suggests that TZ complex proteins may make up the Y-links connecting the axonemal doublets to the membrane [21, 22].

However, as each of the studies found different components to the complexes, there is no consensus on the exact composition of each complex, their formation, the contribution of each component, or their mechanism of function as ciliary gatekeepers. One possible explanation for this may be tissue- or organism-specific variation in TZ complex composition. While these complexes have been studied in different model organisms, the presence or absence of each gene in each model organism has not been examined. Therefore we set out to provide a detailed examination of the evolutionary patterns of these genes. We present here a comprehensive analysis of presence/absence of 19 TZ complex proteins across 52 eukaryotic organisms, both ciliated and non-ciliated, and identify 6 proteins which are almost ubiquitously present in ciliated eukaryotes and which may represent the core of a dynamic TZ complex.

## Results and discussion

**Figure 2:**
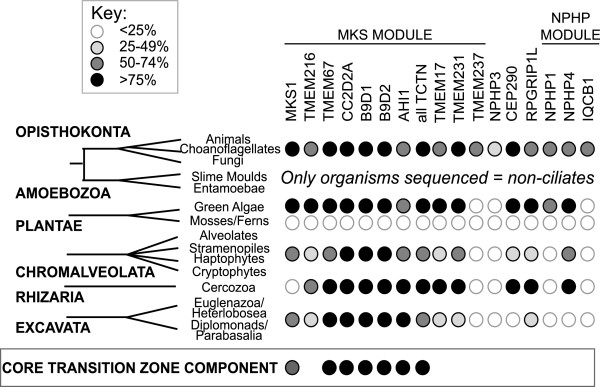
**Evolutionary distribution of ciliary transition zone genes.** Stylised eukaryotic tree showing distribution of TZ complex proteins across 6 eukaryotic supergroups. All non-ciliated organisms have been excluded from the table. Plantae are divided into two sub-groups: green algae and mosses/ferns (higher plants are non-ciliate). Both these sub-groups contain ciliated organisms; note that mosses and ferns lack all known TZ complex components. Black circles denote presence of a putative orthologue in >75% ciliated/flagellate organisms analysed in one supergroup. Dark grey, light grey and white circles represent presence in 50-74%, 25-49% and <25% organisms analysed in one supergroup, respectively. The boxed area shows proteins that are present in >50% organisms in every supergroup; these represent the core TZ components. MKS1 is present in >50% of every supergroup but missing from the Rhizaria, which are represented by only a single sequenced organism; additional genome sequences may indicate that MKS1 is also core. Additional file 2 shows a more detailed version listing individual organisms, and the full bioinformatic analysis is available in Additional file 1.

Thus far, 19 proteins have been identified biochemically as part of protein complexes which maintain ciliary compartmentalisation, though the exact constituents of these complexes in different model systems has not been elucidated and there may well be other components yet to be identified. To identify the complement of TZ complex proteins in different eukaryotes, we carried out a bioinformatic survey of 19 proteins in 52 organisms, including representatives from all six eukaryotic supergroups [25]. Full results, including accession numbers and e-values, can be found in Additional file 1 and these results are summarised in Additional file 2. In common with centriolar proteins [1, 4, 26], we found considerable diversity in the conservation of TZ components across eukaryotes (Figure 2, Additional file 2). All 19 proteins were restricted to eukaryotes which build cilia and flagella (Additional file 2). However, the majority of the TZ proteins had a widespread evolutionary distribution, with the exception of NPHP3, NPHP5/IQCB1 and TMEM237 which were restricted to opisthokonts (animals and fungi). TMEM237 and NPHP3 are notable in that they were not found outside of metazoan organisms, suggesting they may have evolved concomitant with multicellularity.

### Only 6 TZ proteins are conserved across the majority of ciliated eukaryotes

We identified a core group of 6 proteins, consisting of TMEM67, CC2D2A, B9D1, B9D2, AHI1, and a single TCTN family member, which are present in >50% of all ciliate/flagellate organisms analysed in each supergroup (boxed area, Figure 2). This suggests that these six proteins are likely to have critical conserved functions. All are implicated in Meckel-Gruber and/or Joubert syndromes [18, 27–35], two severe ciliopathies, highlighting their importance for cilium function. A 7^th^ protein, MKS1, is present in >50% of five of the six supergroups and is only absent from Rhizaria; this supergroup is currently represented by a single sequenced genome in this analysis, and as more genomes become available it may become apparent that MKS1 is also core.

All six of these core proteins belong to the larger of the two known TZ complexes, the MKS/B9/TCTN complex [17, 18, 20]. Our analysis indicates that while proteins belonging to the MKS complex are consistently found across the six eukaryotic supergroups, proteins which form the NPHP complex (NPHP1, NPHP4 and IQCB1/NPHP5) evolved later. NPHP4 is the most prevalent, but is missing from the Excavata. However, the restriction of NPHP1 and NPHP5/IQCB1 to the Opithokonts suggests that the MKS complex initially existed alone and that the NPHP complex evolved later, perhaps in concordance with increasing complexity or the need for redundancy. As such, a single complex containing NPHP4 may exist outside the opisthokonts, or NPHP4 may have other functions. This may explain why RPGRIP1Lis not one of the core conserved proteins; studies in *C. elegans* have shown that, in this organism at least, the two complexes are redundant with RPGRIP1L the only protein which causes significant ciliary dysfunction upon abrogation [20]. Generally, RPGRIP1L is only present together with at least one component of the NPHP module, usually NPHP4; the exception is the insects, where 2 of the 4 organisms examined (*Anopheles gambiae* and *Apis mellifera*) have an RPGRIP1L orthologue but lack any apparent constituents of the NPHP complex. This implies that the MKS module may be able to assemble in the absence of RPGRIP1L, but the NPHP module cannot.

### Organisms lacking TZ complex components have unusual ciliary biology

Intriguingly, our analysis shows that several ciliated organisms – *Giardia intestinalis, Plasmodium falciparum, Toxoplasma gondii, Physcomitrella patens* and *Selaginella moellendorffii* – apparently lack all (or in the case of *T. gondii*, all but one) of the known TZ complex proteins. There are several unusual aspects to the ciliary biology in these particular organisms which might indicate that they have evolved alternative mechanisms of maintaining ciliary compartmentalisation, or indeed do not require a traditional separation of the ciliary compartment; in many, ultrastructural studies have not yet confirmed whether a TZ is present. Unlike most other organisms, the proximal region of the *Giardia* flagellum is found within the cytoplasm and is not membrane-bound; IFT particles are thought to dock directly onto the sides of the exposed microtubules within the cytoplasm rather than being restricted to the ciliary base as in other model organisms [36]. It is unclear whether the Giardia flagellum contains a TZ region at all, and Y-links have neither been observed ultrastructurally nor would be expected without a surrounding membrane. Giardia may therefore have evolved specific mechanisms to maintain ciliary compartmentalisation. The Apicomplexa (Plasmodium, Toxoplasma) also lack TZ components; this may correlate with a lack of several centriolar proteins in these organisms [3, 37]. *Plasmodium* species lack IFT components in their predicted proteome [3, 37] and therefore build their flagella intracellularly by IFT-independent mechanisms and then extrude them from the cell [38]. Further, flagella in these organisms are restricted to the short-lived male gamete and the presence of a TZ with or without Y-links has not been confirmed. In contrast, several IFT components have been identified in the proteome of the apicomplexan *Toxoplasma* [3, 37], which likewise builds flagella only in the male gamete [39]; this may correlate with the presence of a single TZ protein (B9D1) in this organism, though the flagellar biology of *Toxoplasma* has likewise been little studied and thus the relevance of this finding remains to be elucidated. It may be that apicomplexa have evolved specific mechanisms to maintain compartmentalisation or, given the fleeting nature of their flagella, do not require strict ciliary compartmentalisation. Given the hypothesis that the conoid complex of apicomplexa may have evolved from flagellar components [40], B9D1 may have adapted to function in this region.

### Non-seed land plants lack TZ complex proteins and have no Y-links

In contrast, the flagella of the non-seed land plants have been characterised in detail [reviewed in 41] and contain a classical TZ. We therefore examined the ultrastructure of the TZ of the *Selaginella* gamete and representatives of five major clades of non-seed land plants (Figure 1). The gross ultrastructure of the TZ in the flagellate sperm of these organisms appeared normal, containing a ring of outer doublet microtubules and the typical stellate pattern also seen in the unicellular green alga *Chlamydomonas* and other plants with motile sperm [41]. However, unlike the mammalian motile cilia and trypanosome flagellum which have prominent Y-links, we could see no evidence of any structures linking the outer doublet microtubules to the ciliary membrane (Figure 1). Since the non-seed land plants also lack a number of components of both the IFT-B and BBSome protein groups previously thought to be essential for proper function and compartmentalisation of the eukaryotic cilium [26, 37], this raises questions about the precise nature of the motile cilium in these organisms.

Previous studies have shown that disruption of TZ complexes causes loss of Y-links and/or changes in microxtubule-membrane linkage [20, 23]; as such, the correlation between absence of both TZ complex proteins and ciliary Y-links in the non-seed land plants is consistent with the theory that the TZ complex proteins are involved in the formation of Y-links [21, 22]. Disruption of several TZ complex components affects Y-link formation [20, 23], though as yet, Cep290 is the only protein implicated in Y-link formation that is known to localise specifically to the region between the microtubule doublets and the ciliary membrane [24]. In common with another study [26], our analysis failed to find evidence of a Cep290 homologue in several organisms, including the model organisms *Caenorhabditis elegans* and *Trypanosoma brucei*. Comparative search methods rely on sequence similarity; it is impossible, by these methods, to distinguish between absence of a protein and divergence of that protein to beyond the threshold of detection. In particular, the Cep290 protein sequence consists almost entirely of low-complexity coiled-coil motifs, which confounds sequence comparison methods by producing large numbers of spurious hits against other coiled-coil proteins. Attempts to optimise specificity by further editing the Cep290 protein alignment failed to produce significant improvements in homologue identification. As such, it is likely that additional Cep290 homologues exist beyond those identified here.

### TZ complexes and developmental signalling in animals

While current evidence suggests that TZ complexes are essential to ciliary biology, a role supported by the lack of TZ complex components in non-ciliated organisms and their ubiquity in ciliates, their presence in a specific organism is no guarantee of a role in TZ complexes. Recent studies suggest that TZ complex proteins may function outside of the cilium itself, such as in cytoskeletal organisation [42] and epithelial morphogenesis [43, 44]; further, there is increasing evidence that many ciliopathy proteins may have extra-ciliary roles e.g. [45–48]. Due to the preponderance of phenotypes caused by dysregulation of Hh and Wnt signalling pathways in both model organisms and patients, many studies of ciliopathy proteins have focused on their impact on metazoan developmental signalling pathways. Disruption of several TZ complex proteins leads to significant effects on signalling pathways, such as Wnt signalling in the case of TMEM67/TMEM216/TMEM237 [23, 49–51]. By examining the evolutionary patterns of these groups of proteins, we can gain better understanding of their possible functions. For example, TMEM67 has been proposed to be a receptor in the Wnt signalling pathway [52], with TMEM216 and TMEM237 acting as modifiers to its function in this regard [23, 42]. Our data indicate that TMEM67 is one of the core six proteins found across the majority of ciliated eukaryotes, while TMEM216 is less frequently found. Therefore TMEM67 likely evolved prior to TMEM216 and, at least originally, functioned without it. Further, TMEM237 is found only in the metazoa, suggesting that it may have evolved as an additional regulator of TMEM67 concomitant with multicellularity.

It is also important to note that both classical Hh and Wnt signalling are restricted largely to metazoan organisms [53], though there is evidence of a primitive form of Wnt signalling in the amoebozoa [54]. In contrast, TZ complex proteins are found throughout eukaryotic evolution. This supports the theory that they may impact upon developmental signalling pathways indirectly by affecting ciliary permeability, preventing the correct passage of signalling intermediates between the ciliary and cytoplasmic compartments. The sheer diversity of TZ complex proteins in metazoa suggests that these proteins may have diversified in function, but the absence of any TZ complex proteins in non-ciliates implies that the ancestral role of these proteins was ciliary.

## Conclusions

With all of this in mind, there may be a minimal set of proteins required to correctly compartmentalise the cilium. We would predict, based on the domain architecture of the core 6 proteins [22], that the B9 domain has a critical function, and that membrane anchoring via TMEM and C2 domains is key. How this is then connected to the microtubules is not clear, but several TZ complex proteins localise to the centrosome in non-ciliated cells e.g. [55–58]. Given the near-ubiquity of TMEM67, CC2D2A, B9D1, B9D2, AHI1, a single TCTN family member, and possibly MKS1 across ciliated eukaryotes, we suggest a dynamic complex built around these proteins and varying in a spatio-temporal and/or organism/tissue-specific manner. In concordance with current ideas [21, 22], formation of this complex is likely to be key in the proper formation and functioning of ciliary Y-links. To further examine the relationships between these complexes and their constituent protein members, it will be important to fully integrate metazoan models with single-celled organisms to properly examine the molecular nature of complex formation and to properly disentangle effects on ciliary compartmentalisation from the potentially diverse effects caused by changes in developmental signalling pathways and non-ciliary roles. By clarifying the evolutionary relationships between TZ complex proteins and the protein complements of various model organisms, we hope to provide a valuable tool in the understanding how these complexes might work in different organisms and tissues essential to both basic cilium and ciliopathy research.

## Methods

### Bioinformatic analysis of TZ complex proteins

Proteins previously identified as members of TZ complexes were selected from previous literature [17–20, 23] and the following *Homo sapiens* RefSeq protein sequences identified as the longest isoform of each human homologue: MKS1 [NCBI:NP_060247.2], TMEM216 [NCBI:NP_001167462.1], TMEM67 [NCBI:NP_714915.3], CEP290 [NCBI:NP_079390.3], RPGRIP1L [NCBI:NP_056087.2], CC2D2A [NCBI:NP_001073991.2], B9D1 [NCBI:NP_001230402.1], B9D2 [NCBI:NP_085055.2], AHI1 [NCBI:NP_001128303.1], TCTN1 [NCBI:NP_001076007.1], TCTN2 [NCBI:NP_079085.2], TCTN3 [NCBI:NP_056446.4], TMEM17 [NCBI:NP_938017.2], TMEM231 [NCBI:NP_001070884.1], TMEM237 [NCBI:NP_001037850.1], NPHP1 [NCBI:NP_000263.2], NPHP3 [NCBI:NP_694972.3], NPHP4 [NCBI:NP_055917.1], IQCB1/NPHP5 [NCBI:NP_001018864.2]. Septin2 was excluded due to a high level of sequence similarity to other Septin family members, making differentiation between different septins in divergent organisms difficult. RPGRIP1 and RPGRIP1L are paralogous sequences that are 31% identical and 43% similar at the protein level in humans, and are readily distinguishable in metazoans using the search methods in this paper. Non-metazoan organisms have only a single sequence, which is more similar to RPGRIP1L. Only RPGRIP1L has been identified biochemically in TZ complexes involved in ciliary compartmentalisation, and thus only RPGRIP1L is included here. Finally, as all causative genes for the severe ciliopathy Meckel-Gruber Syndrome (MKS) are known complex members with the exception of NPHP3 (MKS7), this was included for completeness.

The selected protein sequences were used to query the non-redundant predicted proteomes of 52 organisms (40 flagellate, 12 non-flagellate) chosen to represent a wide evolutionary spread of eukaryotes. A list of proteome sources and versions is included in Additional file 1: Table S1. BLASTp and reciprocal BLASTp searches [59] were carried out using the NCBI stand-alone BLAST + application version 2.2.25+ [60] against BLAST-formatted proteomes. Any resultant hits with an e-value below 1e-05 were confirmed by reciprocal BLAST against the *Homo sapiens* genome, and considered putative orthologues if the original sequence was returned. Hits with an e-value below 1e-25 (with the exception of MKS2, where an e-value of 1e-20 was used) were aligned using MAFFT [61, 62], and columns containing more than 50% gaps removed to prevent species- or clade-specific insertions biasing the results. This alignment was then used either as a query alignment for PSI-BLAST [59], or to generate a HMM using HMMer v3.0 (hmmer.org, [63]) which was subsequently used as a query for HMM searches. The e-value thresholds for these methods were 1e-10 (PSI-BLAST) and 1e-15 (HMM).

Some proteins contained domains or motifs which produced a large number of false positives, e.g. the WD40 domain of AHI1. To remove these confounding hits these proteins modified prior to search, and consist of the following: AHI1 (amino acids 1–545 only), NPHP3 (animo acids 210–606 only), NPHP1 (amino acid 219-end only) and IQCB1 (IQ domains removed). Further, it was often difficult to distinguish between TCTN1, 2 and 3 due to high levels of sequence similarity; the same hit/s were often returned from any of the three query sequences with varying e-values. As such, an alignment using all TCTN proteins found by BLASTp with a e-value better than 1e-25 was generated and used as a query for PSI-BLAST; a HMM was further generated from this alignment and used as a query for HMM searches. From these data, each organism was scored as to the total number of TCTN proteins found.

To be included as a putative orthologue (green or blue boxes, Additional file 1: Table S1), proteins with an e-value better than cut-off must have returned the original starting sequence when queried against the Homo sapiens proteome and, if close to the cut-off value (e-value within 5 orders of magnitude) further confirmed by examination of alignments generated by MAFFT and domain identification via InterProScan [64, 65]. If a hit did not pass this curation process, the result was considered the same as no orthologue (red boxes, Additional file 1: Table S1). In the case of CEP290, coiled-coil motifs found throughout the protein introduced a number of false positive hits but could not be removed; these false positives affected PSI-BLAST searches only and resulted in a large number of hits better than cut-off being discarded.

### Transmission electron microscopy

Cells were fixed in 2.5% glutaraldehyde, 2% paraformaldehyde and 0.1% picric acid in 100 mM phosphate pH 6.5 (for fixation of *T. brucei*), or 100 mM sodium cacodylate pH 7.0 (IMCD3 cells or rat primary tracheal cells), post-fixed in 1% osmium tetroxide and stained en bloc with 2% aqueous uranyl acetate. Land plant materials were fixed in 3-6% glutaraldehyde in 0.05 M Pipes buffer (pH 7.4) for 2–4 h, washed in 0.1 M cacodylate buffer (pH 7.2) and postfixed in 2% OsO_4._ Following dehydration through a graded series of acetone and propylene oxide, the material was embedded in Epon resin and cured at 60°C for 16 h prior to sectioning. Thin sections were poststained for 5 min each with 2% ethanolic uranyl acetate and basic lead citrate.

### Availability of supporting data

The data set supporting the results of this article is included within the article in Additional file 1. This includes all accession numbers, e-values and genome sources.

## Electronic supplementary material

Additional file 1: **Bioinformatic analysis of TZ complex components.** Table showing the full bioinformatic analysis including accession numbers and e-values. Dark green indicates a predicted orthologue, blue indicates a possible orthologue (found by less than 3 search methods or only partially supported by manual curation – see supplementary methods for details), red indicates no orthologue. For the TCTN proteins it was difficult to distinguish between TCTN1, 2, and 3. Dark green indicates the most likely orthologue as supported by e-values, but often the same hit was returned from all 3 query sequences (light green). The combined PSI-BLAST and HMM TCTN results are displayed as a separate column (see supplementary methods). Presence of 1 TCTN is coloured light green, presence of more than 1 TCTN is coloured dark green. Red indicates no orthologue found. Core TZ proteins are highlighted in grey. (PDF 236 KB)

Additional file 2: **Evolutionary patterns of TZ complex components.** Summary table showing presence (black) or absence (white) of a predicted orthologue in each organism. Grey denotes a possible orthologue. It was difficult to distinguish between TCTN proteins in most organisms; the boxed area indicates the presence of 2 or more TCTNs (black circles), 1 TCTN (grey circles) or no TCTN (white circles). 3 TCTN proteins are only present in vertebrates. (PDF 891 KB)
