# Supplementary material for: Bioinformatic analysis of ciliary transition zone proteins reveals insights into the evolution of ciliopathy networks
Source: BMC Genomics. 2014 Jun 26;15(1):531. doi: 10.1186/1471-2164-15-531 (PMC4092220; doi:10.1186/1471-2164-15-531)
Supplement: Supplementary file 1 — Additional file 1: Bioinformatic analysis of TZ complex components. Table showing the full bioinformatic analysis including accession numbers and e-values. Dark green indicates a predicted orthologue, blue indicates a possible orthologue (found by less than 3 search methods or only partially supported by manual curation – see supplementary methods for details), red indicates no orthologue. For the TCTN proteins it was difficult to distinguish between TCTN1, 2, and 3. Dark green indicates the most likely orthologue as supported by e-values, but often the same hit was returned from all 3 query sequences (light green). The combined PSI-BLAST and HMM TCTN results are displayed as a separate column (see supplementary methods). Presence of 1 TCTN is coloured light green, presence of more than 1 TCTN is coloured dark green. Red indicates no orthologue found. Core TZ proteins are highlighted in grey. (PDF 236 KB) [file 12864_2014_6210_MOESM1_ESM.pdf]

Organism	Genome Source & Version	Phylogeny	Motile Cilia	Immotile Cilia	MKS1	TMEM216	TMEM67
Acyrthosiphon pisum	NCBI (Build 2.0)	Opisthokonta/Metazoa/Arthropoda/Hexapoda/Insecta	?		XP_003245424.1 (8.0E-31; 3.0E-137; 1.8E-116)	3.8; 0.002; 8.9E-08	XP_001942698.2 , XP_003247566.1 (1E-145, 2E-25; 0.0, 4E-72; 0, 5.2E-64)
Amphimedon queenslandica	NCBI (Build 1.1)	Opisthokonta/Metazoa/Porifera			XP_003387793.1 (1.0E-27; 2.0E-73; 2.7E-109)	XP_003387222.1 (1.0E-11; 8.0E-14; 5.5E-31)	XP_003389575.1, XP_003391466.1, XP_003390617.1 (N/A, 3E-09, 3E-07; 6E-15, 7E-17, 2E-10; 3.4E-35, 1.4E-26, 1.7E-17)
Anopheles gambiae	Ensembl (AgamP3)	Opisthokonta/Metazoa/Arthropoda/Hexapoda/Insecta			AGAP004735-PA (2.0E-14; 2.0E-36; 2.0E-40)	AGAP003795-PA (5.0E-10; 2.0E-14; 4.7E-27)	AGAP002548-PA (3.0E-83; 3.0E-174; 1.2E-242)
Apis mellifera	NCBI (build 5.0)	Opisthokonta/Metazoa/Arthropoda/Hexapoda/Insecta			Only hits above cutoff MKS9 & 10 (1.9; 0.05; NO HITS)	0.86; 2.1; NO HITS	0.34; 4.0E-05; 0.25
Arabidopsis thaliana	NCBI (Build consists of TAIR10 & ASM21127v1)	Plantae/Viridiplantae/Angiosperms			0.02; 1.1; NO HITS	NO HITS; 4.5; NO HITS	1.5; 0.25; 0.027
Aspergillus niger	JGI (v3.0)	Opisthokonta/Fungi/Dikarya			2.2; 0.57; NO HITS	1.1; 3.8; NO HITS	NO HITS; 0.24; 0.05
Aureococcus anophagefferens	JGI (v1.0)	Chromalveolata/Stramenophiles (heterokonts)	Centriole/flagella genes in genome, but no known flagellate stage & acentriolar in life cycle stages analysed to date		3.8; 0.43; NO HITS	0.25; 3.0E-05; 1.2E-06	61468, 22040 (2E-59, 6E-14; 8eE-179, 2E-17; 1.6E-215, 8E-28)
Batrachochytrium dendrobatidis	JGI (v1.0)	Opisthokonta/Fungi/Chytridiomycota			33942 (7.0E-28; 2.0E-77; 7.7E-100)	0.013; 4.00E-04; 2.1E-007	89929 (2.0E-61; 1.0E-139; 8.8E-200)
Bigelowiella natans	JGI (v1.0)	Rhizaria/Cercozoa			Only hits above cutoff MKS9 & 10 (0.32; 3.2; NO HITS)	61960 – reciprocal BLASTs to TMEM80. (2.0E-11; 2.0E-15; 1.7E-29)	86971 (9.0E-64; 6.0E-143; 1.2E-245)
Branchiostoma floridae	JGI (v1.0)	Opisthokonta/Metazoa/Chordata/Cephalocordata			279949 (2.0E-156; 4.0E-149; 5.8E-225)	276936 (4.0E-29; 1.0E-32; 3.1E-63)	98238, 106148 (0.0, 0.0; 0.0, 0.0; 0.0, 0.0)
Brugia malayi	WormBase (Dec 2010 Release)	Opisthokonta/Metazoa/Pseudocoelomata/Nemotoda			BM19460_BMAL6908.1_BMAL6908.1 (2.0E-07; 5.0E-16; 1.5E-36)	0.35; 0.81; NO HITS	BM02106_Bm1_22085A_BMAL3855.1, BM17103_BMAL3855.1_BMAL3855.1, BM09272_BMAL11508.1_BMAL11508.1 (4E-93,3E-92, 9E-19; 5E-174, 1E-172, 3E-32; 5.1E-229,8.9E-229, 7.4E-43)
Caenorhabditis elegans	WormBase (Release CB4)	Opisthokonta/Metazoa/Pseudocoelomata/Nemotoda			R148.1a_CE40712_WBGene00020100, R148.1b_CE41329_WBGene00020100 (2E-10, 3E-10; 3E-23, 5E-23; 4.0E-24, 2.8E-24)	WBGene00194710 (8.0E-12; 6.0E-10; 7.8E-22)	[illegible]
Capitella sp.	JGI (v1.0)	Opisthokonta/Metazoa/Coelomata/Protostomia/Annelida			157534 (5.0E-124; 6.0E-149; 5.3E-214)	180232 (2.0E-24; 4.0E-30; 4.3E-59)	[illegible]
Chlamydomonas reinhardtii	Phytozome (v4.3)	Viridiplantae/Chlorophyta/Chlorophyceae			Cre12.g495350.t1.2 (1.0E-34; 8.0E-94; 1.2E-160)	Cre08.g379750.t1.1 (6.0E-22; 8.0E-37; 3.9E-59)	[illegible]
Chlorella sp.	JGI (v1.0)	Viridiplantae/Chlorophyta/Trebouxiophyceae	Flagella genes in genome, but no flagellate stage known		4.5; NO HITS; NO HITS	0.003; 2.1; NO HITS	[illegible]
Ciona intestinalis	NCBI (Build 1.0), JGI (v2.0)	Opisthokonta/Metazoa/Coelomata/			270563, XP_002121395.1, 230876 (4E-113, 4E-106, 5E-24; 3E-137, 2E-133, 4E-44; 5.1E-191, 8.4E-186, 3.1E-71)	286465, XP_002126879.1 (8E-22, 1E-21; 2E-25, 2E-25; 3.6E-55, 3.5E-55)	[illegible]
Cyanidioschyzon merolae	*Cyanidioschyzon merolae* Genome	Viridaplantae/Rhodophyta/Bangiophyceae/Cyanidiales			0.41; 0.82; NO HITS	NO HITS; 0.036; NO HITS	[illegible]
Danio rerio	NCBI (Build 5)	Opisthokonta/Metazoa/Chordata/Craniata			NP_001070841.2 (0.0; 8.0E-147; 5.0E-219)	XP_683769.2 (3.0E-36; 2.0E-31; 1.4E-62)	[illegible]
Daphnia pulex	JGI (v1.0)	Opisthokonta/Metazoa/Arthropoda/Crustacea			111648 (1.0E-24; 1.0E-39; 1.0E-51)	5; NO HITS; 0.001	[illegible]
Dictyostelium discoideum	JGI (v1.0)	Amoebozoa/Mycetozoa			3.80; 0.07; NO HITS	0.75; 2.3; NO HITS	[illegible]
Drosophila melanogaster	FlyBase (Release 5.42)	Opisthokonta/Metazoa/Arthropoda/Hexapoda/Insecta			FBpp0073500 (4.0E-15; 5.0E-32; 4.1E-48)	[FBpp0289771, FBpp0289770 & FBpp0081388], FBpp0081390 (1E-10, 7E-09; 2E-14, 2E-12; 1.4E-28, 1.3E-24)	[illegible]
Emiliania huxleyi	JGI (v1.0)	Chromalveolata/Haptophyta			1.40; 3.0E-06; 2.0E-13	222967 (7.0E-11; 5.0E-11; 1.9E-26)	[illegible]
Entamoeba histolytica	AmoebaDB (Release 1.6)	Amoebozoa/Archamoebae			4.70; 0.03; NO HITS	NO HITS; 0.80; NO HITS	[illegible]
Gallus gallus	NCBI (Build 3.1)	Opisthokonta/Metazoa/Chordata/Craniata/Vertebrata/Euteleostomi/Archosauria			XP_415705.2 (0.0; 6.0E-140; 1.2E-195)	XP_001233295.1, XP_001236892.1 (1E-19, 1E-21; 1E-18, 3E-15; 3.2E-41, 1E-38)	[illegible]
Giardia intestinalis	GiardiaDB (Release 2.5)	Excavata/Diplomonadida			0.07; 0.02; NO HITS	1.5; 3.5; NO HITS	[illegible]
Guillardia theta	JGI (v1.0)	Chromalveolata/Cryptophyta			121078 (3.0E-34; 9.0E-90; 4.1E-101)	150704 (1.0E-19; 1.0E-20; 1.3E-39)	[illegible]
Helobdella robusta	JGI (v1.0)	Opisthokonta/Metazoa/Annelida			N/A; 0.81; 2.1E-05	62996 (7.0E-05; 2.0E-06; 8.9E-18)	[illegible]
Homo sapiens	NCBI (Build 37.3)	Opisthokonta/Metazoa/Chordata/Craniata/Vertebrata/Euteleostomi/Mammalia			NP_060247.2, NP_001159399.1 (0.0, 0.0; 1E-146, 3E-139; 2E-218, 6.9E-210)	NP_001167462.1, NP_001167461.1 (1E-79, 4e-76; 8E-31, 2E-30; 8.3E-61, 1.9E-60)	[illegible]
Hydra magnapapillata	NCBI (Build 1)	Opisthokonta/Metazoa/Cnidaria		?	XP_002157754.1 (5.0E-82; 6.0E-133; 1.8E-179)	NO HITS; 8.2; NO HITS	[illegible]
Laccaria bicolor	JGI (v2.0)	Opisthokonta/Fungi/Dikarya			3.2; 1.3; NO HITS	0.13; 6.5; NO HITS	[illegible]
Lottia gigantea	JGI (v1.0)	Opisthokonta/Metazoa/Mollusca			228399 (5.0E-125; 2.0E-144; 1.1E-215)	204590 (2.0E-22; 2.0E-32; 1.1E-61)	[illegible]
Micromonas pusilla	JGI (v3.0)	Viridiplantae/Chlorophyta/Mamiellophyceae			209829 (9.0E-35; 5.0E-90; 5.8E-131)	206136, 196083 (2E-12, 3E-07; 1E-16, 9E-16; 2.7E-30, 1.1E-17)	[illegible]
Monosiga brevicollis	JGI (v1.0)	Opisthokonta/Choanoflagellida/Codonosigidae			30962 (6.0E-15; 1.0E-32; 1.4E-48)	0.16; 0.007; 5.3E-05	[illegible]
Naegleria gruberi	JGI (v1.0)	Excavata/Heterolobosea/Schizopyrenida			29577 (2.0E-29; 1.0E-47; 5.6E-63)	0.23; 5.0E-11; 2.9E-13	[illegible]
Nematostella vectensis	JGI (v1.0)	Opisthokonta/Metazoa/Cnidaria			209121, 248809 (7E-78, 1E-32; 2E-113, 9E-24; 3.5E-141, 4.9E-45)	175269 (3.0E-24; 3.0E-27; 6.7E-57)	[illegible]
Paramecium tetraurelia	ParameciumDB (v1.61)	Chromalveolata/Alveolata/Ciliophora/Intramacronucleata			GSPATG00011904001 (2.0E-19; 5.0E-33; 3.6E-48)	2E-11, 2E-11, 4E-11; 7E-15, 1E-14, 1E-14; 2E-29, 7.4E-29, 8.9E-29	[illegible]
Phaeodactylum tricornutum	JGI (v2.0)	Chromalveolata/Stramenophiles (heterokonts)			1.1; 0.49; NO HITS	0.66; NO HITS; NO HITS	[illegible]
Physcomitrella patens	JGI (v1.1)	Viridiplantae/Streptophyta/Embryophyta			7.7; 0.46; NO HITS	0.14; 5.1; NO HITS	[illegible]
Plasmodium falciparum	PlasmoDB (v8.1)	Chromalveolata/Alveolata/Apicomplexa			1.2; 0.52; NO HITS	0.27; 0.53; NO HITS	[illegible]
Populus trichocarpa	JGI (v1.1)	Viridiplantae/Streptophyta/Embryophyta			0.17; 0.15; NO HITS	NO HITS; NO HITS; NO HITS	[illegible]
Saccharomyces cerevisiae	SGD (R64-1-1)	Opisthokonta/Fungi/Dikarya			0.4; 0.84; NO HITS	4.4; 1.2; NO HITS	[illegible]
Schistosoma mansoni	GeneDB (v4.0)	Opisthokonta/Metazoa/Platyhelminthes			Smp_049610 (4.0E-21; 9.0E-28; 1.7E-45)	Smp_114590 (0.018; 5.0E-07; 1.8E-15)	[illegible]
Selaginella moellendorffii	JGI (v1.0)	Viridiplantae/Streptophyta/Embryophyta			0.55; 1.6; NO HITS	NO HITS; 2.2; 0.049	[illegible]
Strongylocentrotus purpuratus	NCBI (Build 3.1)	Opisthokonta/Metazoa/Echinodermata			XP_787637.2 (7.0E-133; 3.0E-140; 4.0E-219)	XP_799067.2, XP_796540.3 (2E-28, 3E-28; 1E-31, 8E-32; 5.6E-54, 4.3E-54)	[illegible]
Tetrahymena thermophila	TGD (Oct2008 Release)	Chromalveolata/alveolata/Ciliophora/Intramacronucleata			2441_m00056 (6.0E-19; 3.0E-32; 1.8E-48)	2; 2.8; NO HITS	[illegible]
Thalassiosira pseudonana	JGI (v3.0)	Chromoalveolata/Stramenophiles			5008 (1.0E-05; 6.0E-13; 5.6E-18)	0.16; NO HITS; NO HITS	[illegible]
Toxoplasma gondii	ToxoDB (Release 7.1)	Chromalveolata/Alveolata/Apicomplexa			3.6; 1.4; NO HITS	0.71; 0.57; 0.023	[illegible]
Tribolium casteneum	NCBI (Build 2)	Opisthokonta/Metazoa/Arthropoda/Hexapodia/Insecta			XP_001813387.1 (4.0E-56; 3.0E-116; 1.1E-144)	XP_968423.1 (7.0E-09; 5.0E-16; 5.6E-29)	[illegible]
Trichomonas vaginalis	TrichDB (Release 1.3)	Excavata/Parabasalia			TVAG_028180, TVAG_166340 (1E-17, 1E-17; 2E-41, 2E-41; 1.6E-44, 1.1E-44)	TVAG_062950 (1.0E-04; 2.0E-08; 4.9E-20)	[illegible]
Trichoplax adhaerens	JGI (v1.0)	Opisthokonta/Metazoa/Placozoa		?	61316 (6.0E-105; 8.0E-131; 1.4E-198)	1.3; 6.3; NO HITS	[illegible]
Trypanosoma brucei	TriTrypDB (Release 3.3)	Excavata/Euglemozoa/Kinetoplastidae			Tb927.8.3430 (0.001; 1.0E-09; 7.9E-09)	Tb927.4.3710 (0.003; 7.0E-005; 3.8E-010)	[illegible]
Ustilago maydis	JGI (v1.0)	Opisthokonta/Fungi/Dikarya			0.29; 0.51; NO HITS	2.4; 2.9; NO HITS	[illegible]

Organism	CEP290	RPGRIP1L	CC2D2A	B9D1	B9D2	AHI1	TCTN1	TCTN2	TCTN3	TCTN (1, 2, 3 combined)	TMEM17	TMEM231	TMEM237	NPHP1	NPHP3	NPHP4	IQCB1
Acyrthosiphon pisum	[illegible]	[illegible]	[illegible]	[illegible]	[illegible]	[illegible] (3.0E-08; 1.0E-18; 9.3E-23)	XP_001947098.2 (7.0E-15; 2.0E-44; 3.5E-45)	XP_001947098.2 (8.0E-04; 2.0E-15; 4.1E-18)	XP_001947098.2 (1.0E-20; 1.0E-51; 2.6E-63)	XP_001947098.2 (6.00E-54; 3.60E-72)	XP_001952768.1 (2.0E-29; 2.0E-47; 9.9E-70)	XP_001948207.2 (3.0E-36; 5.0E-89; 2.2E-107)	0.4; 1.0E-09; 8.1E-08	0.92; 0.16; 5.2E-04	0.1; 1.0E-04; 0.0077	7; 0.003; 0.0049	0.017; 4.0E-06; NO HITS
Amphimedon queenslandica	[illegible]	[illegible]	[illegible]	[illegible]	[illegible]	0.87; 0.011; 0.0025	XP_003387537.1 (1.0E-11; 9.0E-42; 1.4E-56)	XP_003387537.1 (3.0E-05; 1.0E-13; 5.9E-35)	XP_003387537.1 (8.0E-21; 4.0E-56; 6.8E-86)	XP_003387537.1 (1.0E-41; 2.8E-88)	XP_003389447.1 (8.0E-21; 2.0E-20; 2.1E-43)	XP_003384386.1 (3.0E-33; 2.0E-81; 8.3E-108)	0.081; 1.0E-04; 0.086	1.1; 0.95; 1.2E-07	0.53; 5.0E-05; 0.0032	0.19; 6.0E-06; 4.7	0.003; 2.0E-05; NO HITS
Anopheles gambiae	[illegible]	[illegible]	[illegible]	[illegible]	[illegible]	5.7; 0.019; 0.069	0.96; 0.33; NO HITS	0.012; 3.0E-08; N/A	1.0E-06; 0.18; NO HITS	0.35; NO HITS	0.002; 0.057; 2.8E-06	AGAP008571-PA (0.003; 6.0E-13; 9.1E-21)	0.79; 3.0E-04; 0.044	1.6; 0.2; 7.2E-06	0.49; 4.0E-05; 0.0047	0.4; 0.001; 0.028	0.005; 8.0E-07; 5.0E-04
Apis mellifera	[illegible]	[illegible]	[illegible]	[illegible]	[illegible]	[illegible]	[illegible]	[illegible]	[illegible]	[illegible]	[illegible]	[illegible]	[illegible]	[illegible]	[illegible]	[illegible]	[illegible]
Arabidopsis thaliana	[illegible]	[illegible]	[illegible]	[illegible]	[illegible]	[illegible]	[illegible]	[illegible]	[illegible]	[illegible]	[illegible]	[illegible]	[illegible]	[illegible]	[illegible]	[illegible]	[illegible]
Aspergillus niger	[illegible]	[illegible]	[illegible]	[illegible]	[illegible]	[illegible]	[illegible]	[illegible]	[illegible]	[illegible]	[illegible]	[illegible]	[illegible]	[illegible]	[illegible]	[illegible]	[illegible]
Aureococcus anophagefferens	[illegible]	[illegible]	[illegible]	[illegible]	[illegible]	[illegible]	[illegible]	[illegible]	[illegible]	[illegible]	[illegible]	[illegible]	[illegible]	[illegible]	[illegible]	[illegible]	[illegible]
Batrachochytrium dendrobatidis	[illegible]	[illegible]	[illegible]	[illegible]	[illegible]	[illegible]	[illegible]	[illegible]	[illegible]	[illegible]	[illegible]	[illegible]	[illegible]	[illegible]	[illegible]	[illegible]	[illegible]
Bigelowiella natans	[illegible]	[illegible]	[illegible]	[illegible]	[illegible]	[illegible]	[illegible]	[illegible]	[illegible]	[illegible]	[illegible]	[illegible]	[illegible]	[illegible]	[illegible]	[illegible]	[illegible]
Branchiostoma floridae	[illegible]	[illegible]	[illegible]	[illegible]	[illegible]	[illegible]	[illegible]	[illegible]	[illegible]	[illegible]	[illegible]	[illegible]	[illegible]	[illegible]	[illegible]	[illegible]	[illegible]
Brugia malayi	[illegible]	[illegible]	[illegible]	[illegible]	[illegible]	[illegible]	[illegible]	[illegible]	[illegible]	[illegible]	[illegible]	[illegible]	[illegible]	[illegible]	[illegible]	[illegible]	[illegible]
Caenorhabditis elegans	[illegible]	[illegible]	[illegible]	[illegible]	[illegible]	[illegible]	[illegible]	[illegible]	[illegible]	[illegible]	[illegible]	[illegible]	[illegible]	[illegible]	[illegible]	[illegible]	[illegible]
Capitella sp.	[illegible]	[illegible]	[illegible]	[illegible]	[illegible]	[illegible]	[illegible]	[illegible]	[illegible]	[illegible]	[illegible]	[illegible]	[illegible]	[illegible]	[illegible]	[illegible]	[illegible]
Chlamydomonas reinhardtii	[illegible]	[illegible]	[illegible]	[illegible]	[illegible]	[illegible]	[illegible]	[illegible]	[illegible]	[illegible]	[illegible]	[illegible]	[illegible]	[illegible]	[illegible]	[illegible]	[illegible]
Chlorella sp.	[illegible]	[illegible]	[illegible]	[illegible]	[illegible]	[illegible]	[illegible]	[illegible]	[illegible]	[illegible]	[illegible]	[illegible]	[illegible]	[illegible]	[illegible]	[illegible]	[illegible]
Ciona intestinalis	[illegible]	[illegible]	[illegible]	[illegible]	[illegible]	[illegible]	[illegible]	[illegible]	[illegible]	[illegible]	[illegible]	[illegible]	[illegible]	[illegible]	[illegible]	[illegible]	[illegible]
Cyanidioschyzon merolae	[illegible]	[illegible]	[illegible]	[illegible]	[illegible]	[illegible]	[illegible]	[illegible]	[illegible]	[illegible]	[illegible]	[illegible]	[illegible]	[illegible]	[illegible]	[illegible]	[illegible]
Danio rerio	[illegible]	[illegible]	[illegible]	[illegible]	[illegible]	[illegible]	[illegible]	[illegible]	[illegible]	[illegible]	[illegible]	[illegible]	[illegible]	[illegible]	[illegible]	[illegible]	[illegible]
Daphnia pulex	[illegible]	[illegible]	[illegible]	[illegible]	[illegible]	[illegible]	[illegible]	[illegible]	[illegible]	[illegible]	[illegible]	[illegible]	[illegible]	[illegible]	[illegible]	[illegible]	[illegible]
Dictyostelium discoideum	[illegible]	[illegible]	[illegible]	[illegible]	[illegible]	[illegible]	[illegible]	[illegible]	[illegible]	[illegible]	[illegible]	[illegible]	[illegible]	[illegible]	[illegible]	[illegible]	[illegible]
Drosophila melanogaster	[illegible]	[illegible]	[illegible]	[illegible]	[illegible]	[illegible]	[illegible]	[illegible]	[illegible]	[illegible]	[illegible]	[illegible]	[illegible]	[illegible]	[illegible]	[illegible]	[illegible]
Emiliania huxleyi	[illegible]	[illegible]	[illegible]	[illegible]	[illegible]	[illegible]	[illegible]	[illegible]	[illegible]	[illegible]	[illegible]	[illegible]	[illegible]	[illegible]	[illegible]	[illegible]	[illegible]
Entamoeba histolytica	[illegible]	[illegible]	[illegible]	[illegible]	[illegible]	[illegible]	[illegible]	[illegible]	[illegible]	[illegible]	[illegible]	[illegible]	[illegible]	[illegible]	[illegible]	[illegible]	[illegible]
Gallus gallus	[illegible]	[illegible]	[illegible]	[illegible]	[illegible]	[illegible]	[illegible]	[illegible]	[illegible]	[illegible]	[illegible]	[illegible]	[illegible]	[illegible]	[illegible]	[illegible]	[illegible]
Giardia intestinalis	[illegible]	[illegible]	[illegible]	[illegible]	[illegible]	[illegible]	[illegible]	[illegible]	[illegible]	[illegible]	[illegible]	[illegible]	[illegible]	[illegible]	[illegible]	[illegible]	[illegible]
Guillardia theta	[illegible]	[illegible]	[illegible]	[illegible]	[illegible]	[illegible]	[illegible]	[illegible]	[illegible]	[illegible]	[illegible]	[illegible]	[illegible]	[illegible]	[illegible]	[illegible]	[illegible]
Helobdella robusta	[illegible]	[illegible]	[illegible]	[illegible]	[illegible]	[illegible]	[illegible]	[illegible]	[illegible]	[illegible]	[illegible]	[illegible]	[illegible]	[illegible]	[illegible]	[illegible]	[illegible]
Homo sapiens	[illegible]	[illegible]	[illegible]	[illegible]	[illegible]	[illegible]	[illegible]	[illegible]	[illegible]	[illegible]	[illegible]	[illegible]	[illegible]	[illegible]	[illegible]	[illegible]	[illegible]
Hydra magnapapillata	[illegible]	[illegible]	[illegible]	[illegible]	[illegible]	[illegible]	[illegible]	[illegible]	[illegible]	[illegible]	[illegible]	[illegible]	[illegible]	[illegible]	[illegible]	[illegible]	[illegible]
Laccaria bicolor	[illegible]	[illegible]	[illegible]	[illegible]	[illegible]	[illegible]	[illegible]	[illegible]	[illegible]	[illegible]	[illegible]	[illegible]	[illegible]	[illegible]	[illegible]	[illegible]	[illegible]
Lottia gigantea	[illegible]	[illegible]	[illegible]	[illegible]	[illegible]	[illegible]	[illegible]	[illegible]	[illegible]	[illegible]	[illegible]	[illegible]	[illegible]	[illegible]	[illegible]	[illegible]	[illegible]
Micromonas pusilla	[illegible]	[illegible]	[illegible]	[illegible]	[illegible]	[illegible]	[illegible]	[illegible]	[illegible]	[illegible]	[illegible]	[illegible]	[illegible]	[illegible]	[illegible]	[illegible]	[illegible]
Monosiga brevicollis	[illegible]	[illegible]	[illegible]	[illegible]	[illegible]	[illegible]	[illegible]	[illegible]	[illegible]	[illegible]	[illegible]	[illegible]	[illegible]	[illegible]	[illegible]	[illegible]	[illegible]
Naegleria gruberi	[illegible]	[illegible]	[illegible]	[illegible]	[illegible]	[illegible]	[illegible]	[illegible]	[illegible]	[illegible]	[illegible]	[illegible]	[illegible]	[illegible]	[illegible]	[illegible]	[illegible]
Nematostella vectensis	[illegible]	[illegible]	[illegible]	[illegible]	[illegible]	[illegible]	[illegible]	[illegible]	[illegible]	[illegible]	[illegible]	[illegible]	[illegible]	[illegible]	[illegible]	[illegible]	[illegible]
Paramecium tetraurelia	[illegible]	[illegible]	[illegible]	[illegible]	[illegible]	[illegible]	[illegible]	[illegible]	[illegible]	[illegible]	[illegible]	[illegible]	[illegible]	[illegible]	[illegible]	[illegible]	[illegible]
Phaeodactylum tricornutum	[illegible]	[illegible]	[illegible]	[illegible]	[illegible]	[illegible]	[illegible]	[illegible]	[illegible]	[illegible]	[illegible]	[illegible]	[illegible]	[illegible]	[illegible]	[illegible]	[illegible]
Physcomitrella patens	[illegible]	[illegible]	[illegible]	[illegible]	[illegible]	[illegible]	[illegible]	[illegible]	[illegible]	[illegible]	[illegible]	[illegible]	[illegible]	[illegible]	[illegible]	[illegible]	[illegible]
Plasmodium falciparum	[illegible]	[illegible]	[illegible]	[illegible]	[illegible]	[illegible]	[illegible]	[illegible]	[illegible]	[illegible]	[illegible]	[illegible]	[illegible]	[illegible]	[illegible]	[illegible]	[illegible]
Populus trichocarpa	[illegible]	[illegible]	[illegible]	[illegible]	[illegible]	[illegible]	[illegible]	[illegible]	[illegible]	[illegible]	[illegible]	[illegible]	[illegible]	[illegible]	[illegible]	[illegible]	[illegible]
Saccharomyces cerevisiae	[illegible]	[illegible]	[illegible]	[illegible]	[illegible]	[illegible]	[illegible]	[illegible]	[illegible]	[illegible]	[illegible]	[illegible]	[illegible]	[illegible]	[illegible]	[illegible]	[illegible]
Schistosoma mansoni	[illegible]	[illegible]	[illegible]	[illegible]	[illegible]	[illegible]	[illegible]	[illegible]	[illegible]	[illegible]	[illegible]	[illegible]	[illegible]	[illegible]	[illegible]	[illegible]	[illegible]
Selaginella moellendorffii	[illegible]	[illegible]	[illegible]	[illegible]	[illegible]	[illegible]	[illegible]	[illegible]	[illegible]	[illegible]	[illegible]	[illegible]	[illegible]	[illegible]	[illegible]	[illegible]	[illegible]
Strongylocentrotus purpuratus	[illegible]	[illegible]	[illegible]	[illegible]	[illegible]	[illegible]	[illegible]	[illegible]	[illegible]	[illegible]	[illegible]	[illegible]	[illegible]	[illegible]	[illegible]	[illegible]	[illegible]
Tetrahymena thermophila	[illegible]	[illegible]	[illegible]	[illegible]	[illegible]	[illegible]	[illegible]	[illegible]	[illegible]	[illegible]	[illegible]	[illegible]	[illegible]	[illegible]	[illegible]	[illegible]	[illegible]
Thalassiosira pseudonana	[illegible]	[illegible]	[illegible]	[illegible]	[illegible]	[illegible]	[illegible]	[illegible]	[illegible]	[illegible]	[illegible]	[illegible]	[illegible]	[illegible]	[illegible]	[illegible]	[illegible]
Toxoplasma gondii	[illegible]	[illegible]	[illegible]	[illegible]	[illegible]	[illegible]	[illegible]	[illegible]	[illegible]	[illegible]	[illegible]	[illegible]	[illegible]	[illegible]	[illegible]	[illegible]	[illegible]
Tribolium casteneum	[illegible]	[illegible]	[illegible]	[illegible]	[illegible]	[illegible]	[illegible]	[illegible]	[illegible]	[illegible]	[illegible]	[illegible]	[illegible]	[illegible]	[illegible]	[illegible]	[illegible]
Trichomonas vaginalis	[illegible]	[illegible]	[illegible]	[illegible]	[illegible]	[illegible]	[illegible]	[illegible]	[illegible]	[illegible]	[illegible]	[illegible]	[illegible]	[illegible]	[illegible]	[illegible]	[illegible]
Trichoplax adhaerens	[illegible]	[illegible]	[illegible]	[illegible]	[illegible]	[illegible]	[illegible]	[illegible]	[illegible]	[illegible]	[illegible]	[illegible]	[illegible]	[illegible]	[illegible]	[illegible]	[illegible]
Trypanosoma brucei	[illegible]	[illegible]	[illegible]	[illegible]	[illegible]	[illegible]	[illegible]	[illegible]	[illegible]	[illegible]	[illegible]	[illegible]	[illegible]	[illegible]	[illegible]	[illegible]	[illegible]
Ustilago maydis	[illegible]	[illegible]	[illegible]	[illegible]	[illegible]	[illegible]	[illegible]	[illegible]	[illegible]	[illegible]	[illegible]	[illegible]	[illegible]	[illegible]	[illegible]	[illegible]	[illegible]
