# Supplementary figures and images for: Bioinformatic analysis of ciliary transition zone proteins reveals insights into the evolution of ciliopathy networks
Source: BMC Genomics. 2014 Jun 26;15(1):531. doi: 10.1186/1471-2164-15-531 (PMC4092220; doi:10.1186/1471-2164-15-531)

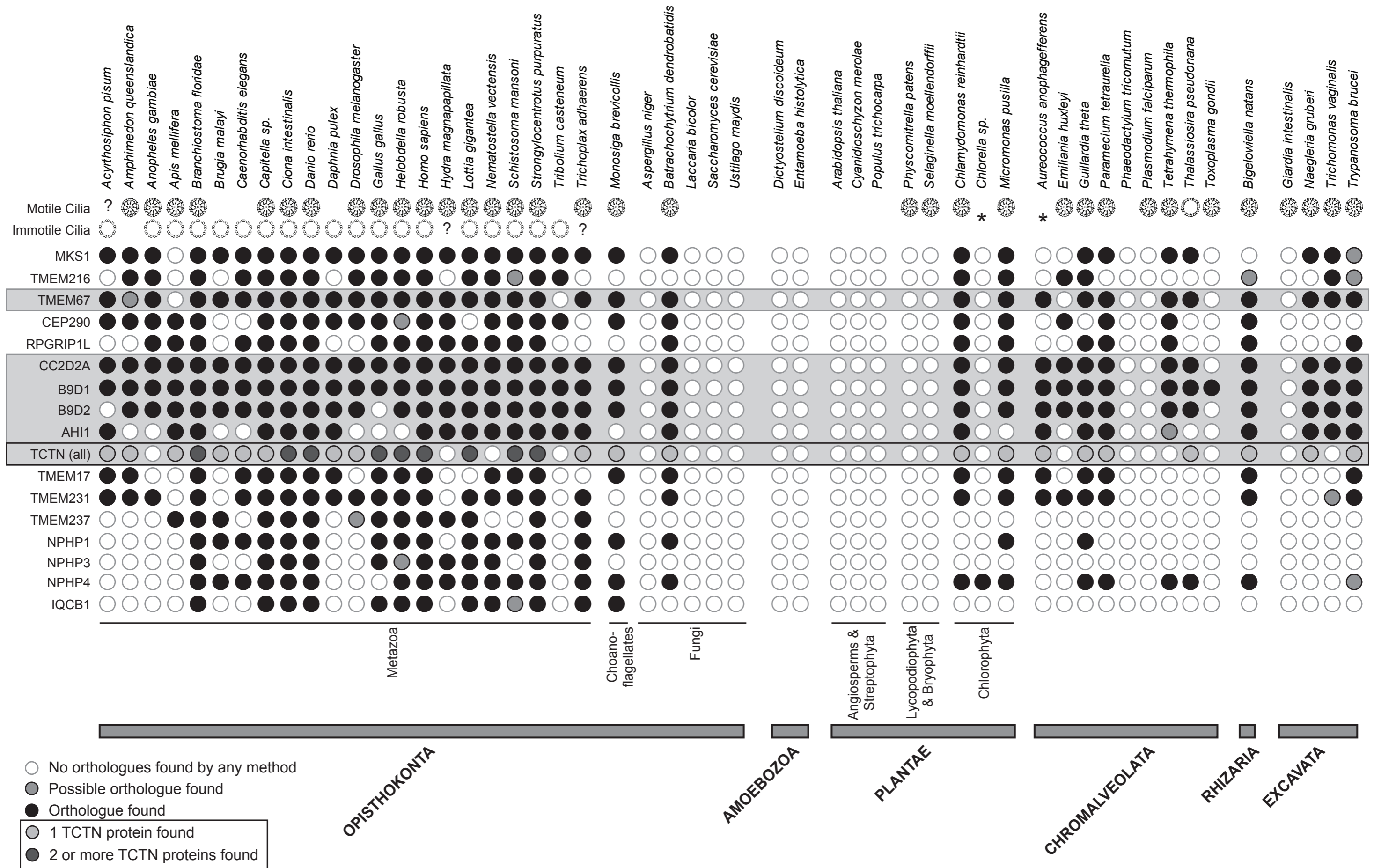

Supplement: Supplementary file 2 — Additional file 2: Evolutionary patterns of TZ complex components. Summary table showing presence (black) or absence (white) of a predicted orthologue in each organism. Grey denotes a possible orthologue. It was difficult to distinguish between TCTN proteins in most organisms; the boxed area indicates the presence of 2 or more TCTNs (black circles), 1 TCTN (grey circles) or no TCTN (white circles). 3 TCTN proteins are only present in vertebrates. (PDF 891 KB) [file 12864_2014_6210_MOESM2_ESM.pdf]
